# Metachronous ovarian endometrioid carcinomas in a patient with a *PTEN* variant: case report of incidentally detected Cowden syndrome

**DOI:** 10.1186/s12885-019-6272-2

**Published:** 2019-10-29

**Authors:** Hiroyuki Matsubayashi, Satomi Higashigawa, Yoshimi Kiyozumi, Yasue Horiuchi, Yasuyuki Hirashima, Nobuhiro Kado, Masato Abe, Takuma Ohishi, Sumiko Ohnami, Kenichi Urakami, Ken Yamaguchi

**Affiliations:** 1 Division of Genetic Medicine Promotion, Shizuoka Cancer Center, Nagaizumi, Suntogun, Shizuoka, 411-8777 Japan; 2 Division of Endoscopy, Shizuoka Cancer Center, 1007 Shimonagakubo, Nagaizumi, Suntogun, Shizuoka, Japan; 3 Division of Gynecology Shizuoka Cancer Center, Nagaizumi, Suntogun, Shizuoka, 411-8777 Japan; 4 Division of Pathology Shizuoka Cancer Center, Nagaizumi, Suntogun, Shizuoka, 411-8777 Japan; 5 Research Institution of Shizuoka Cancer Center, Nagaizumi, Suntogun, Shizuoka, 411-8777 Japan

**Keywords:** Ovarian, Endometrioid carcinoma, PTEN, Germline variant

## Abstract

**Background:**

Somatic *PTEN* mutation occurs in a proportion of ovarian endometrioid carcinomas. However, these cancers have seldom been reported in diseases associated with germline *PTEN* variants, such as Cowden syndrome (CS).

**Case presentation:**

The present case was a 39-year-old woman with a left ovarian carcinoma who demonstrated a germline splice variant of *PTEN* (c.1026 + 1G > T) following genome-wide whole exome sequencing of her germline DNA. Histology of her resected tumor revealed endometrioid carcinoma of the same type as a right ovarian cancer resected eight years previously. These tumors showed null immunostaining for PTEN. She was genetically diagnosed with CS. Despite her clinical examinations had demonstrated several characteristic findings of CS, including mammary fibroma, esophageal and skin papilloma, colonic hamartoma, uterine myoma, and lipoma, the clinicians could not approach this diagnosis.

**Conclusion:**

Ovarian endometrioid carcinoma is generally thought to develop from endometrial tissue menstruated from the uterus and implanted on the ovary. To date, ovarian cancers have not been listed as CS-related cancers; however, ovarian endometrioid cancer can have a potential association with CS in endometriosis cases.

## Background

Pathogenic variants of germline *PTEN* result in autosomal dominant hereditary disease or PTEN hamartoma tumor syndromes (PHTS), including Proteus syndrome (PS), Proteus-like syndrome (PLS), Bannayan-Riley-Ruvalcaba syndrome (BRRS), and Cowden syndrome (CS). CS is clinically diagnosed based on the major criteria associated with three types of cancer (breast, thyroid, and endometrium) and macrocephaly, as well as by minor criteria that include other benign thyroid lesions; intellectual disability (IQ ≤75); intestinal hamartomatous polyps; mammary fibrocystic disease; lipomas; fibromas; genitourinary tumors and malformation; and uterine fibroids [1].

Ovarian cancer is not included in the above criteria, but uterine endometrial carcinoma is included. Ovarian endometrioid carcinoma is thought to develop from endometrial tissue menstruated from the uterus and implanted on the ovary [2]. We report a unique case of CS who had a germline *PTEN* splice variant and subsequently developed metachronous bilateral ovarian endometrioid carcinomas without expressing the PTEN protein.

## Case presentation

**Fig. 1:**
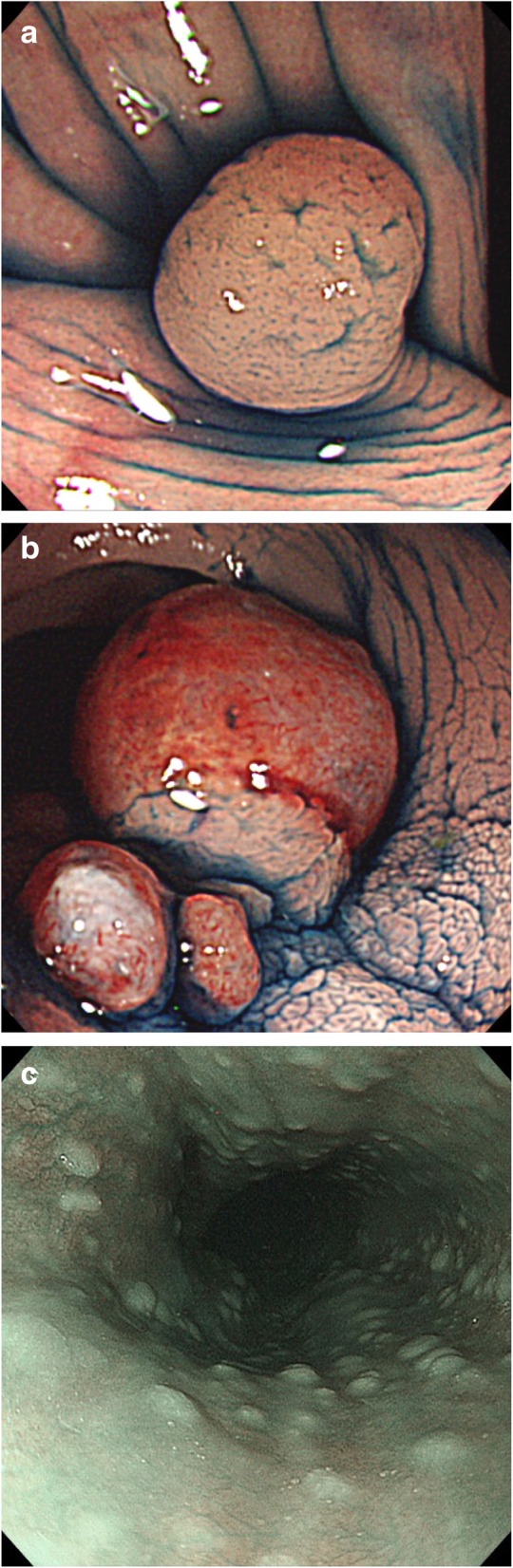
Endoscopic findings. **a** An indigo carmine stained colonic hamartomatous polyp, **b** Indigo carmine stained rectal polyps of heterotopic endometriosis, **c** Screening by upper gastrointestinal endoscopy demonstrated multiple esophageal papillomas and glycogenic acanthosis

The patient was a Japanese woman with a past history of left ovarian endometrioid carcinoma (grade 2, FIGO stage IC1) that had been resected by salpingo-oophorectomy, omentectomy and retroperitoneal lymph node biopsy (fertility sparing surgery) when she was 31 years old. She had a history of multiple thyroid goiters (maximum 2 cm), bilateral breast fibroma treated by surgical resection at age 15, endometriosis (at age 28) and myoma, and salpingitis treated by peroral medications (at age 28). She was intellectual enough to evaluate her IQ over 75. While in her 30s, she sometimes complained of lower abdominal pain and melena. A colonoscopy revealed colorectal polypoid lesions that were histologically diagnosed by forceps biopsy as hamartoma polyps and ectopic endometrial implants (Fig. 1a, b). An upper gastrointestinal endoscopy screening demonstrated multiple esophageal papillomas and glycogenic acanthosis (Fig. 1c). A gluteal subcutaneous lipoma, 55 mm in size, was detected by screening magnetic resonance imaging (MRI). The patient’s mother died of breast cancer in her 40s, and her father had a pathology-confirmed cutaneous papilloma on his head and neck. The patient had no smoking or drinking habits.

**Fig. 2:**
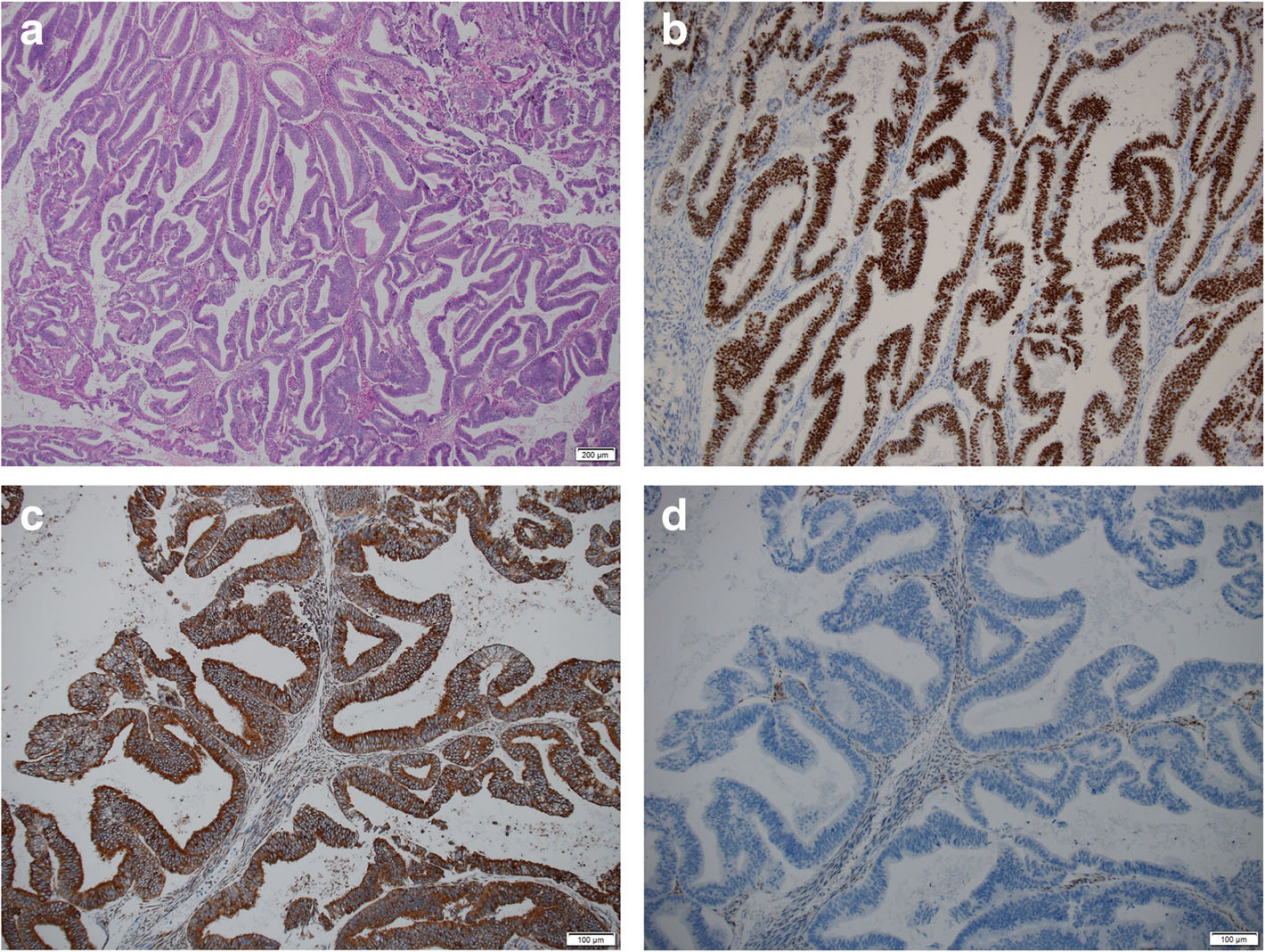
Pathology of the right-ovarian tumor. **a** Hematoxylin and eosin (H&E) staining showing endometrioid carcinoma, grade 1 (× 40). Immunostaining of the tumor showing diffuse expression of estrogen receptor (× 100)(**b**) and PI3K (× 100)(**c**). **d** Null expression of PTEN in the tumor, contrasting with the positive expression in the interstitial cells and inflammatory cells (× 100)

The patient had been undergoing surveillance for the endometriosis by yearly pelvic image examinations, and a right ovarian tumor was detected at age 39. Computed tomography (CT) demonstrated a heterogeneously enhanced mass, 9 cm in size, while ^18^F-fluorodeoxyglucose-positron emission tomography (FDG-PET) showed abnormal uptake by the ovarian tumor (SUVmax: 8.33). She underwent abdominal total hysterectomy, right salpingo-oophorectomy, pelvic lymphadenectomy and para-aortic lymphadenectomy. Endometrioid carcinoma (grade 1, FIGO stage: IC2), partially accompanied with components of squamous metaplasia and low-grade adenofibroma, was detected in the resected ovary. Immunostainings of the right-ovarian endometrial carcinoma revealed high expression of estrogen receptor, PI3K, and Ki-67 (labeling index approximately 40%), but TP53 showed no overexpression, and PTEN, WT1, Napsin A, and HNF-1β were not expressed (Fig. 2). Immunostainings of PI3K showed diffuse expression, but PTEN was not expressed, as also determined in the left ovarian tumor.

All resected lymph nodes were negative for cancer (0/120). Her uterus specimen confirmed myomas and endometriosis.

**Fig. 3:**
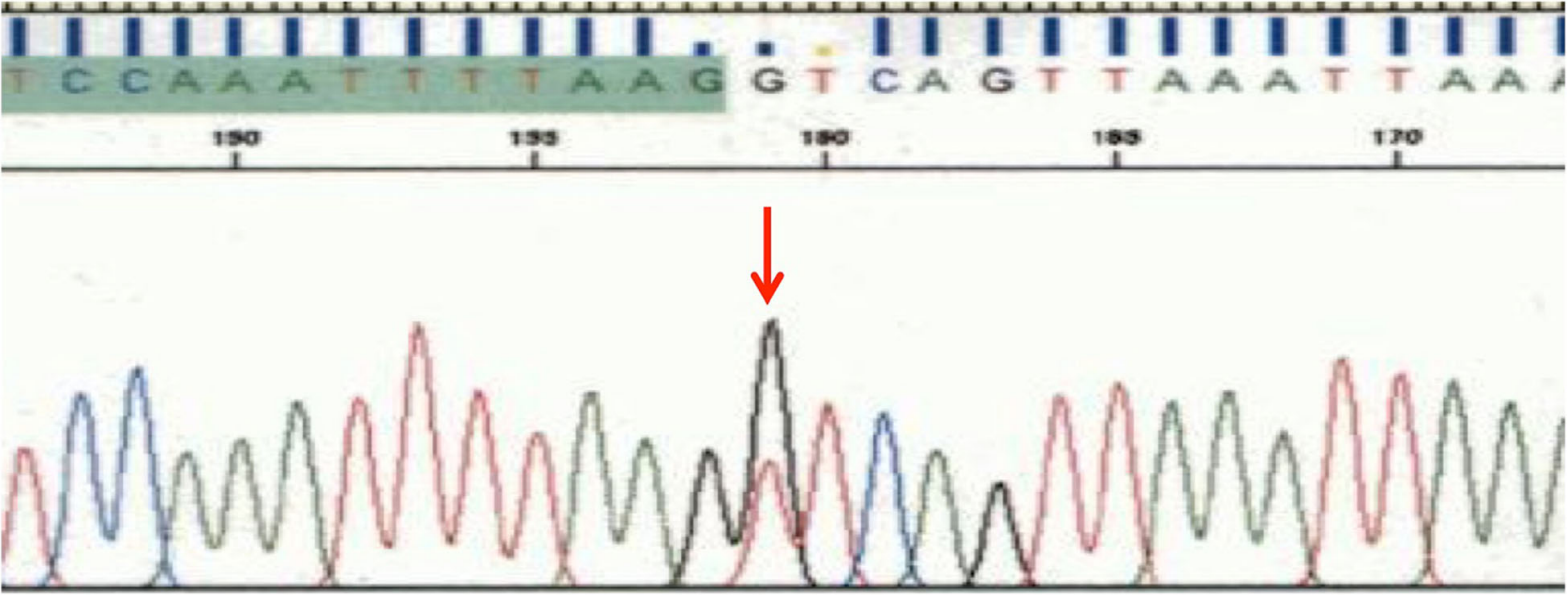
A *PTEN* variant (c.1026 + 1G > T) confirmed by Sanger sequencing (an arrow indicating the variant)

Upon full informed consent, the patient participated in genetic research at the time of surgery. Peripheral blood and a 4–5 mm^3^ area of fresh right-ovarian cancer tissue were taken at surgery for whole exome sequencing (WES) to compare germline DNA and cancer DNA using a next-generation sequencer [3, 4]. An exome library, including 723 cancer-associated genes and 49 genes [3] responsible for hereditary cancer syndromes, was prepared using the Ion AmpliSeq Exome RDY kit (Thermo Fisher Scientific, Massachusetts, USA). Exome sequencing of the right endometrioid carcinoma revealed a pathogenic mutation of hexose-6-phosphate dehydrogenase/glucose 1-dehydrogenase (*H6PD*), *TFAP2D*, *MYO7A*, *TGM1*, *SEMA6B*, *ZNF99*, and *SIGLEC1*. Loss of heterozygosity (LOH) in the 10q.23.3 (*PTEN*) locus was not recognized by copy number variation analysis in this tumor DNA. Her blood DNA demonstrated a splice-donor site variant of *PTEN* (c.1026 + 1G > T) [5], that had been reported to be causative of intellectual disability. This sequence was confirmed by the Sanger sequencing (Fig. 3). No other germline mutation or incidental pathogenic finding was recognized.

Despite a number of characteristic findings in her demographics, images and pathologies, the clinicians did not approach the diagnosis of Cowden syndrome (CS) until the detection of germline *PTEN* variant. At the counseling, multiple (> 6) palmar small papules, buccal papillomas, and macrocephaly (head circumference: 60 cm, > 97th percentile) were further recognized by the physical examinations, fully meeting the clinical criteria of CS. She received clinical surveillance of the organs associated with PHTS [1]. Her father also underwent genetic test, but the variant of *PTEN* was not recognized. Three years have passed since the last surgery. No recurrence has been detected.

## Discussion and conclusions

An elevated risk of ovarian carcinoma has not been reported in cases with germline *PTEN* variants, although an association of *PTEN* with uterine endometrial carcinoma is well known (standardized incidence rate: 42.9, lifetime risk: 28.2%) [6]. However, ovarian endometrioid carcinoma is thought to develop from endometrial tissue menstruated in retrograde and implanted on the ovary [2]. Somatic mutation of *PTEN* is recognized in 53% of endometriosis cases [7], and LOH in the 10q.23.3 locus occurs in a range from 25% [8] to 84% [7]. Moreover, *PTEN* mutation is recognized in 20% [9, 10] of ovarian endometrioid carcinomas and the LOH is 60 [10] to 64% [8]; however, these values are much lower in other types of ovarian carcinomas (2% mutation rate; 28% LOH) [10]. These data suggest a specific association of *PTEN* alterations and endometrioid-type ovarian cancer.

Ovarian endometrioid carcinoma has seldom been reported in cases of Cowden syndrome (CS). The current case demonstrated metachronous bilateral ovarian endometrioid carcinoma with an 8-year interval. Endometriosis, but not endometrioid carcinoma, was recognized in the patient’s resected uterus; therefore, we speculated that an independent carcinogenesis had occurred in the bilateral ovaries or that carcinoma cells had exfoliated from the unilateral ovary and implanted to the other side. The expression patterns of PI3K and PTEN were the same; however, metastasis (from the left to the right ovary) had not been anticipated since both tumors contained low-grade components, which rarely metastasize. Our case suggested a risk for non-uterine endometrioid carcinoma in cases of CS, indicating that all ectopic endometrioses are at risk and require careful follow up or endoscopic resection of rectal endometriosis. Most recently, a case of CS was reported from France, that carried a pathogenic *PTEN* variant (c.388C > T) and developed an ovarian clear cell carcinoma showing loss of PTEN expression [11], like our case. Ovarian clear cell carcinoma has several biological characteristics similar to those of endometrioid carcinoma, such as a pathogenesis through the ovarian endometriosis and common somatic mutations [*PIK3CA* (15–60%), *ARID1A* (30–57%), *CTNNB1* (3–53%) and DNA mismatch repair genes (4–13%)] [9, 12]. In addition to these endometriosis-associated ovarian cancers, several cases of embryonic tumors [13] and dysgerminoma [14] have also reported in cases of CS with germline *PTEN* variants, however their pathological and genetic rationale is unclear. These data give us a warning for the ovarian findings including endometriosis in cases of CS [15].

The patient had several characteristic CS findings that satisfied one major criteria (macrocephaly) and five minor criteria (multiple thyroid goiters, bilateral mammary fibromas, intestinal hamartomas, an uterine myoma, and a gluteal lipoma), so she could be clinically diagnosed with CS [1]. Based on the retrospective assessment using Cleveland Clinic PTEN Risk Calculator (http://www.lerner.ccf.org/gmi/ccscore/), probability of *PTEN* variant in this patient was estimated between 99.4 and 100%, even not including her histories of ovarian endometrioid cancers. However, she had not been diagnosed until after the genetic test for several reasons: the lack of a sense of inherited diseases in the doctor’s mind, multi-department associations beyond each doctor’s expertise field, diagnostic criteria that were characteristic but not really specific to CS, and an atypical CS cancer type. The patient did not have any children, so familial information was limited to her mother who had died of breast cancer at her 40s. In this sense, clinical cancer sequencing using next generation sequencing would potentially increase the possibility of incidentally detecting inherited cancer syndromes with atypical findings or relatively weak phenotypes.

Clinical sequencing sometimes reveals a novel variant that needs determination of its pathogenicity. The current splice donor site variant (c.1026 + 1G > T) is located at the intron 8 or C-terminal site of *PTEN,* and its pathogenicity has been reported previously for characteristic PTEN intellectual disability [5]. To the best of our knowledge, the current report is the first cancer case associated with this *PTEN* variant (c.1026 + 1G > T), and the cancer was negative for PTEN expression, as confirmed by the immunostaining antibody, clone 6H2.1, that recognizes an epitope located at the C-terminal region. PHTS has a genotype–phenotype correlation and includes a cancer-associated (CS and BRRS) and a non-cancer associated (PS and PLS) [1] group. Some *PTEN* variants only cause cancers but show no other criteria of CS [4]. In this sense, the current pathogenic variant of *PTEN* (c.1026 + 1G > T) appears to associate with a variety of characteristic CS findings.

We reported an incidentally detected case of CS in a patient who had carried a pathogenic variant of *PTEN* (c.1026 + 1G > T) and developed bilateral ovarian endometrioid cancers without PTEN expression. Ovarian endometrioid carcinoma can be considered a CS-associated cancer, and caution is needed for ovarian findings including endometriosis in cases of CS.

## Availability of data and materials

The datasets used and/or analyzed in the current are available from the corresponding author on reasonable request.

## Data Availability

All data generated or analyzed during this study are included in this article.

## Abbreviations

BRRS: Bannayan-Riley-Ruvalcaba syndrome
CS: Cowden syndrome
CT: Computed tomography
FDG-PET: ^18^F-fluorodeoxyglucose-positron emission tomography
LOH: Loss of heterozygosity
MRI: Magnetic resonance imaging
PHTS: PTEN hamartoma tumor syndromes
PLS: Proteus-like syndrome
PS: Proteus syndrome
WES: Whole exome sequencing
